# Hepatic Surgical Stress Promotes Systemic Immunothrombosis That Results in Distant Organ Injury

**DOI:** 10.3389/fimmu.2020.00987

**Published:** 2020-05-22

**Authors:** Hongji Zhang, Julie Goswami, Patrick Varley, Dirk J. van der Windt, Jinghua Ren, Patricia Loughran, Hamza Yazdani, Matthew D. Neal, Richard L. Simmons, Jinxiang Zhang, Allan Tsung, Hai Huang

**Affiliations:** ^1^Department of Surgery, Wexner Medical Center, The Ohio State University, Columbus, OH, United States; ^2^Department of Surgery, Union Hospital, Huazhong University of Science and Technology, Wuhan, China; ^3^Department of Surgery, University of Pittsburgh Medical Center, Pittsburgh, PA, United States; ^4^Cancer Center, Union Hospital, Huazhong University of Science and Technology, Wuhan, China; ^5^Department of Cell Biology, Center for Biologic Imaging, University of Pittsburgh Medical Center, Pittsburgh, PA, United States

**Keywords:** neutrophil extracellular traps, immunothrombosis, platelets, liver sterile inflammation, surgical stress

## Abstract

Innate immunity can initiate platelet activation during the development of thrombosis through a process, termed immunothrombosis. Neutrophils form neutrophil extracellular traps (NETs) that have been shown to interact directly with platelets and play pro-coagulant roles in a variety of infectious and sterile inflammatory settings. Hepatic surgical stress initiated by ischemia/reperfusion (I/R) injury has wide systemic consequences on distant organs. However, the mechanisms of this remote injury phenomenon are not well-understood. Here, we sought to determine the role of NETs in causing systemic immunothrombosis and distant organ injury following a local inflammatory insult with liver I/R. Postoperative thromboelastographic revealed that the speed of clot formation (alpha-angle) was significantly increased whereas time to clot formation (R-time) were decreased by in patients undergoing liver resection, indicating a hypercoagulable state after surgery. In mice subjected to liver I/R, circulating platelet activation and platelet-neutrophil aggregates were significantly increased. Injured distant organs such as the lung and kidney displayed NETs and platelet-rich micro-thrombi in the microvasculature following liver I/R. The immune-thrombi and organ damage were dramatically decreased when NETs were inhibited by DNase treatment. Depletion of *Tlr4* on platelets limited NET-induced activation of platelets but had no effect on NET formation. Furthermore, platelet-specific TLR4 KO mice had significantly reduced distant organ injury with decreased circulating platelet activation, platelet-neutrophil aggregates following liver I/R in comparison to their control counterparts. These data establish that after an acute local inflammatory process, NET-activated platelets can lead to a systemic pro-coagulant state with resultant remote organ injury by immunothrombosis.

## Introduction

Multiple molecular and cellular pathways are involved in the normal coagulation of blood, including fibrin producing coagulation cascade and platelet activation (1, 2). Normal coagulation and hemostasis are essential to prevent blood loss after injury or endothelial disruption. However, dysregulation of the normal hemostatic process can contribute to a vast array of common regional thromboembolic events, such as myocardial infarction, stroke, deep vein thrombosis and pulmonary embolism (3, 4). Systemic dissemination of this process can lead to microvascular occlusion and organ dysfunction (5, 6). The mechanisms involved in these systemic events have called attention to the role of evolutionarily conserved links between innate immunity and coagulation, termed immunothrombosis (7–9).

One of the links between innate immunity and coagulation was discovered after the identification of neutrophil extracellular traps (NETs), which form when neutrophils extrude both their cytoplasmic/granular and nuclear contents into the extracellular space (10). During this process, decondensed chromatin filaments decorated with pro-inflammatory proteins provide host defense by capturing microbial pathogens. However, NETs have also been found to be generated by sterile injury and in turn, orchestrate intense inflammatory responses (11).

Traditionally, there is often worry for coagulopathy in patients undergoing liver resection, injury or transplantation due to the concern for depletion of coagulation factors. However, there is mounting evidence that liver injury such as ischemia/reperfusion (I/R) can actually lead to a hypercoagulable state (12, 13). Liver I/R not only occurs in circulatory shock and resuscitation, but it is an inevitable consequence of liver surgery during which hepatic circulation is interrupted indirectly by manipulation or directly by temporary occlusion of the vasculature to control blood loss (14). Severe liver I/R injury is characterized not only by hepatic injury but also by a widespread systemic sterile inflammatory response with accumulation of inflammatory cells in lung and kidney with associated impairment of function (15–17). We have previously focused on the critical role of NETs in augmenting local organ injury in a model of liver I/R (18, 19). However, the systemic role of NETs in mediating microvascular immuno-thrombi in distant organs after liver I/R has not yet been characterized. In this study, we found that NETs exacerbate remote organ injury after liver inflammatory stress through the establishment of a systemic procoagulant state and diffuse microvascular immuno-thrombi.

## Materials and Methods

### Human Patient Samples

Thromboelastography were performed on the blood samples of patients on post-operative day 1 after undergoing liver resection at the University of Pittsburgh Medical Center (Pittsburgh, PA) between September 2015 and May 2016. All indications for surgery, and the characteristics of the background are listed in Supplementary Table 1. Twelve healthy volunteers served as control. All human materials were obtained under an approved Institutional Review Board protocol (MOD08010372-25/PRO08010372) and written consent was received from all participants prior to inclusion.

### Animals

Male wild-type (WT C57BL/6) mice (8–12 weeks old) were purchased from Jackson Laboratories. TLR4 KO, platelet/megakaryocyte specific TLR4 KO (TLR4^loxP/Pf4−Cre^), and TLR4 Flox (TLR4^loxP/loxP^), mice were generated as previously described (20). The LysMeGFP knockin mice were provided by Dr. Thomas Graf (21). The peptidyl arginine deiminase type IV knockout (PAD4^−/−^) mice were bred at our facility (22). Transgenic mice expressing Cre recombinase linked to platelet factor 4 (Pf4) were obtained from Jackson Laboratory. Stud males were bred with Flox females to generate platelet-specific TLR4 KO mice as previously described (23). Genotyping was performed using polymerase chain reaction. Animal protocols were approved by the Animal Care and Use Committee of the University of Pittsburgh and performed in adherence with National Institutes of Health guidelines for use of laboratory animals.

### Liver I/R

The surgical procedure has been described in detail (24). Briefly, the left and median liver lobes were occluded with a microvascular clamp (Fine Science Tools) for 90 min, and reperfusion was initiated by removal of the clamp, the total reperfusion time were 6, 12, and 24 h. In some experiments, to inhibit NET formation, mice received an intra-peritoneal injection of DNase I (5 mg/kg; Roche), mixed in phosphate-buffered saline (PBS) immediately after ischemia and immediately after removing the clamp. Sham animals underwent anesthesia, laparotomy, and exposure of the portal triad without hepatic ischemia.

### Platelet Depletion

Platelets depletion was performed as described previously with penile vein injection of 1 mg/kg low-endotoxin and azide-free anti-CD41 antibody (anti-CD41, BD Biosciences, San Diego, CA), diluted in 200 μL sterile normal saline (0.9% [wt/vol] sodium chloride). Injection of this mixture reduced the number of circulating platelets to <0.05 × 10^6^ value, 1.0–1.2 × 10^6^ published values (23).

### Platelet Isolation, Counting and Transfusion

Mice were sacrificed by exsanguination via cardiac puncture and collection of their entire circulating volume at 6, 12, and 24 h timepoints after reperfusion or sham laparotomy. Sodium citrate (1:9 ratio by volume) and 1 μmol/L prostaglandin E1 (Sigma) were added. Whole blood samples were centrifuged at 260 g for 8 min to isolate platelet-rich plasma (PRP). Pooled PRP was then centrifuged at 740 g for 10 min. Supernatant platelet poor plasma (PPP) was discarded and the platelet pellet was re-suspended in 1 ml sterile Tyrodes buffer. Consistent with prior descriptions, this method led to <0.01% leukocytes in platelet suspension (23, 25). Complete blood counts including platelet counts were obtained using an ADVIA 120 hematology analyzer (Bayer Diagnostics, Tarrytown, NY). Platelets (value = 1.0–1.2 × 10^6^/μL) from 2 same strain donor mice were then diluted with PBS to a volume of 200 μL and transfused via penile vein into recipient mice just before the model (26).

### Platelet Aggregation

Whole blood samples were collected as above and stimulated with 2 μg/ml collagen (Chrono-log). Platelet aggregation was assessed using a Chrono-log aggregometer (Model 700). Analysis was performed using the aggrolink-8 software (Chrono-log) (27).

### Thromboelastography

Platelet function and coagulopathy were measured by Anemoscope 5000 analyzers (Haemonetics, Braintree, MA) for thromboelastography (TEG) analysis which offers a comprehensive coagulation profile: R-time (time to clot formation), K-time (speed of clot formation), alpha angle (rate of clot formation), and maximal amplitude (MA, clot strength). R-time is typically a measure of coagulation factors. MA, on the other hand, is a measure of platelet activity. Whole blood samples were collected (in 1:9 v/v ratio with sodium citrate) by cardiac puncture as above and added to disposable thromboelastography cups containing 2.0 IU of heparinase I and recalcified with CaCl_2_ (20 μl of 0.2 mol/L). Thromboelastography was performed simultaneously on two channels.

### Neutrophil Isolation and *in vitro* NET Formation

Murine neutrophils were isolated from bone marrow of tibias and femurs as previously described (18, 28). A BD Aria Plus high-speed sorter was used to sort neutrophils after incubation with PE-anti-mouse-Ly6G and APC-Cy7-anti-mouse-CD11b (BD Bioscience). To generate NETs, neutrophils were then plated to adhere in coated plates for 1 h before 4 h stimulation with phorbol 12-myristate 13-acetate (PMA, 30 nM; Sigma-Aldrich). Neutrophils re-suspended in RPMI were also stimulated as described above for NETs formation in cell culture dishes. After discarding the supernatant, NETs were harvested in 5 ml of new medium and centrifuged at 300 g for 10 min to pellet intact cells. Then, the supernatant was further centrifuged at 20,000 g for 30 min to pellet NETs. Washed NETs were then resuspended in 1 ml of RPMI 1640 + 1% BSA. Nucleosomes and cell-free DNA were measured in washed NET preparations to confirm the presence of NETs (29).

### Liver, Lung, and Kidney Damage Assessment

Serum alanine aminotransferase (sALT), creatinine and Blood Urea Nitrogen (BUN) levels were measured using the DRI-CHEM 4000 Chemistry Analyzer System (HESKA). Histologic evaluation by H&E staining was used for measuring liver and lung injury. Liver samples were harvested and processed for hematoxylin and eosin (H&E) as described (18). Samples were imaged and scored using the Suzuki methodology by three independent members from the Center for Biologic Imaging (University of Pittsburgh). The tissues were assessed for the amount (%) of inflammation (sinusoidal congestion, cytoplasmic vacuolization, infiltrating inflammatory cells) and necrosis for characterizing liver damage. Two investigators blinded to group assignments analyzed the samples and determined levels of lung injury according to the semi-quantitative scoring system used for measuring lung injury (30). Serum cystatin C levels were measured using enzyme-linked immunosorbent assay (ELISA) for mouse cystatin C (Abcam).

### Immunofluorescent Staining

Liver, lung, and kidney sections were fixed, stained, and imaged using confocal microscopy as previously described (18, 31). Tissues were incubated with specific primary antibodies as follows: Ly6G (2 μg /ml; BD Bioscience; cat no. 560599), citrullinated histone H3 (5 μg/ml, rabbit IgG; Abcam ab5103), CD41 (5 μg/ml, rat IgG; Abcam, ab33661), fibrinogen (2 μg/ml, sheep IgG; Abcam ab61352). Sections were then incubated with Alexa 488-conjugated F-actin phalloidin (1:500, Invitrogen, San Diego, CA, USA) in the presence of the following secondary antibodies depending on the primary antibody pairing: Cy5–conjugated goat anti-rat IgG (1:1000, for anti-CD41 antibody, Jackson Immunoresearch 112-165-167); 488-conjugated goat anti-rat IgG (1:500 for CD41 antibody, Molecular Probes, A11006) for 1 h. A Hoeschst nuclear stain was applied for 30 s and slides were prepared for imaging. Imaging conditions were maintained at identical settings within each antibody-labeling experiment with original gating performed using the negative control. Large area images in X and Y using a Nikon A1 confocal microscope (purchased with 1S10OD019973-01 awarded to Dr. Simon C. Watkins). Quantification was performed using NIS Elements citH3 staining co-localized with Ly6G staining and normalized by area of actin.

### Quantification of NETs

To quantify NETs in mouse serum, a capture enzyme-linked immunosorbent assay for myeloperoxidase (MPO) associated with DNA was performed as described (18, 32). For the capture antibody, a mouse MPO enzyme-linked immunosorbent assay kit (Hycult Biotech; HK210-01) was used according to the manufacturer's directions. A peroxidase-labeled anti-DNA monoclonal antibody (component 2, Cell Death ELISA PLUS, catalog no. 11774424001; Roche) was used. Serum nucleosome quantification was performed using the Cell Death kit.

### Western Blotting

Whole-cell protein lysates from lung and kidney were used for western blotting. Membranes were incubated using citrullinated-histone H3 (1:1000 Abcam 5103) and actin as an internal control.

### Statistical Analysis

The data presented in the figures are mean ± SEM. Group comparisons were performed using analysis of variance and Student *t*-test (GraphPad Prism version 5). For the human subjects' data analysis, we compared the means of normal control and post-operative MPO-DNA serum levels using a paired student's t representing the data graphically with their 95% confidence interval. The baseline characteristics of each group were compared using Chi-square or Fisher's exact tests for categorical variables. *P* < 0.05 was considered statistically significant. In the figures, the standard symbols were used: ^*^P < 0.05, ^**^P < 0.01, and ^***^P < 0.001.

## Results

### Hepatic Surgical Stress Leads to a Systemic Pro-coagulant State Mediated by Platelet Activation

**Figure 1:**
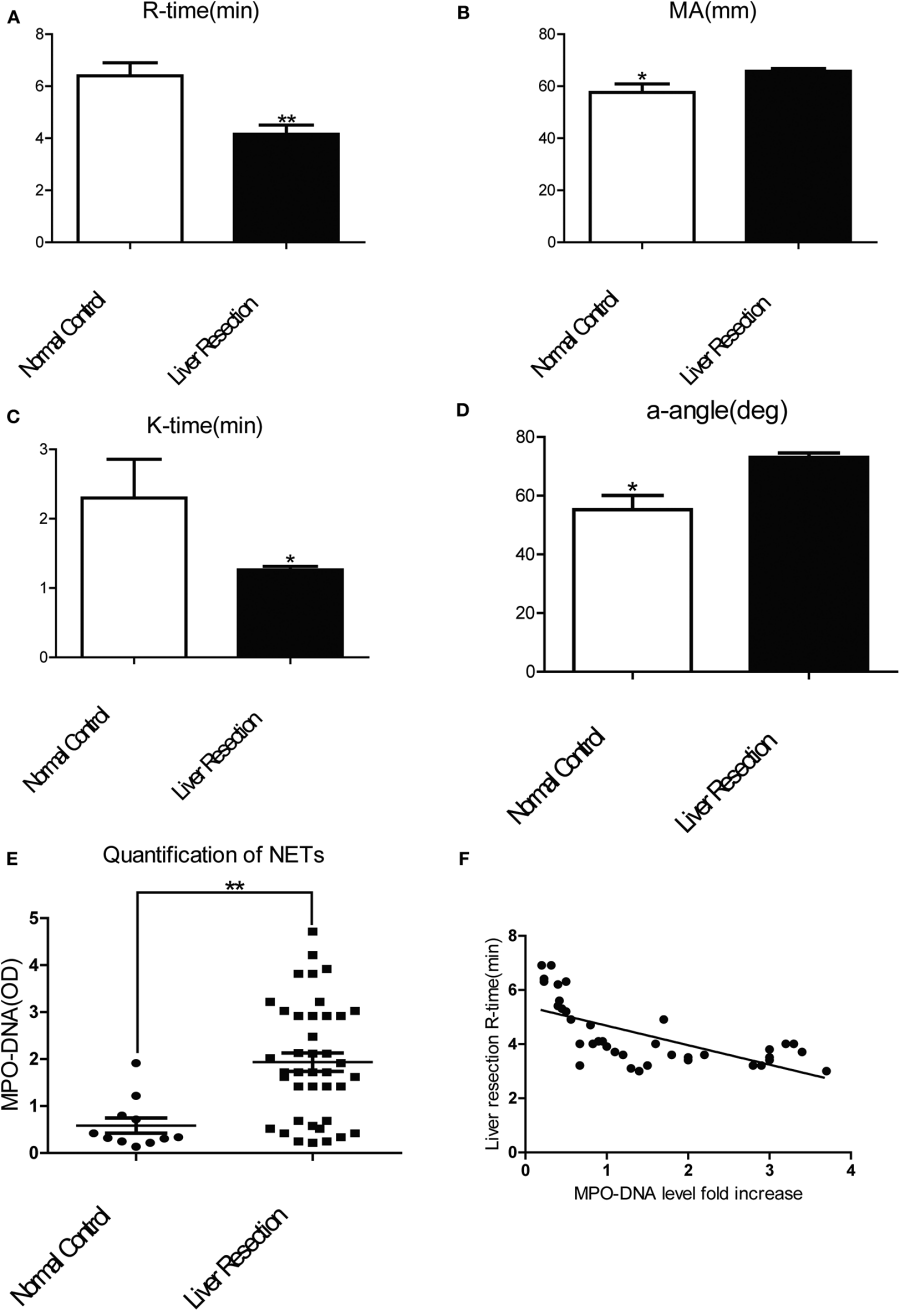
Patients undergoing liver resection are hypercoagulable. Patients undergoing liver resection (*n* = 49) have **(A)** a significant shorter time to clot formation or R-time; **(B)** a greater clot strength or MA; **(C)** a higher speed of clot formation demonstrated by K-time; **(D)** higher rate of clot formation demonstrated by alpha angle; **(E)** increased serum levels of MPO-DNA in comparison to normal control(*n* = 12); **(F)** R-time and serum MPO-DNA levels showed decreased correlation coefficient when measured post-operatively (Spearman's coefficient 0.7, *p* < 0.01). (Abbreviation: Thromboelastogram or TEG: Dynamic change of blood coagulation, R-time: time to clot formation, maximal amplitude (MA): which measures clot strength, K-time: speed of clot formation, α-angle: rate of clot formation, deg: degree). *p < 0.05 and **p < 0.01.

**Figure 2:**
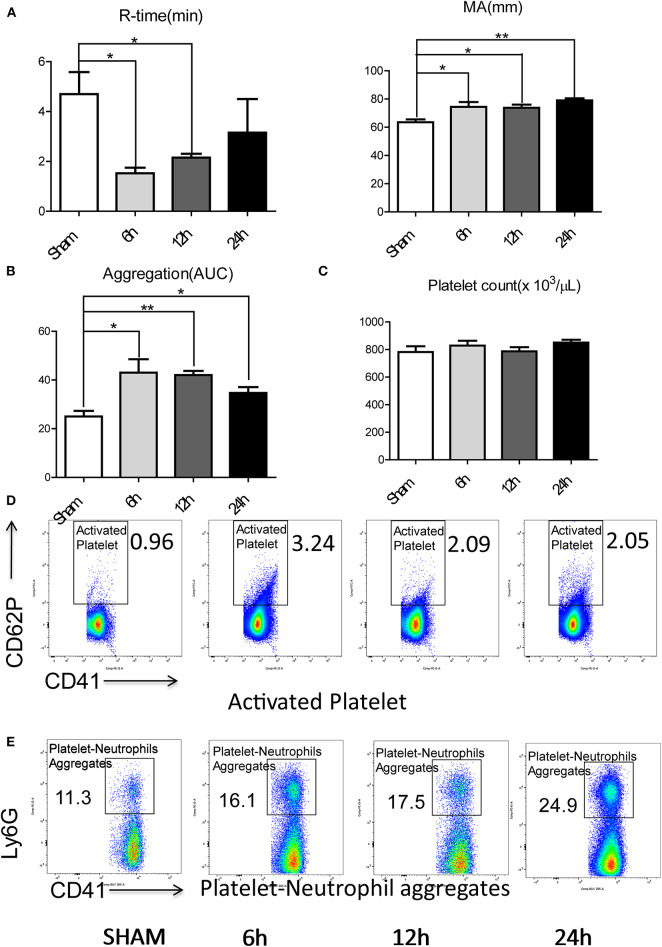
Hepatic surgical stress leads to a systemic pro-coagulant state. Compared with sham mice, IR leads to **(A)** shorter time to clot formation and stronger clot formation; **(B)** greater rate of whole blood aggregation and a greater maximal aggregation; **(C)** consistent platelet counts; **(D)** significant increased platelet activation and **(E)** increased circulating platelet neutrophil aggregates in 6/12/24 h reperfusion after ischemia (*n* = 6–8 each group). (Abbreviation: 6 h: ischemia 1.5 h followed by 6 h reperfusion; 12 h: ischemia 1.5 h followed by 12 h reperfusion; 24 h: ischemia 1.5 h followed by 24 h reperfusion; AUC: area under curve). *p < 0.05 and **p < 0.01.

To investigate if a local hepatic injury could lead to systemic disruptions in coagulation, whole blood samples from 49 human patients between the years 2015–2016, undergoing liver resection and 12 healthy age/gender-matched subjects were collected on postoperatively day 1 for thromboelastography (TEG) analysis (Supplementary Figure 1A). In our cohort of 49 patients (Supplementary Table 1) undergoing liver resection, there was a significantly shorter time to clot formation (R-time) (Figure 1A) and increased clot strength (maximal amplitude, MA) (Figure 1B) in comparison to healthy controls. Furthermore, a higher speed of clot formation demonstrated by K-time (Figure 1C) and a higher rate of clot formation demonstrated by alpha angle (Figure 1D) were observed in these patients compared with healthy controls (33). These data indicated that patients who underwent liver resection had a hypercoagulable status. When neutrophils form NETs, chromatin DNA associated with other neutrophil proteins are released. Consistent with our previous finding, serum MPO-DNA levels, a specific marker of NETs, has been shown to be significantly increased in patients undergoing liver resection compared with healthy controls (Figure 1E) (19, 34). Interestingly, there was a significant correlation between post-operative R-time and serum MPO-DNA complexes, which indicate a correlation between NET formation and hypercoagulation after liver surgery (Figure 1F). We also studied the coagulation changes in our mouse model of hepatic I/R *in vivo* (Supplementary Figure 1B). Consistent with patterns seen in our patient cohort, liver I/R led to significantly shorter R-time and stronger clot formation (MA) (Figure 2A) when compared to animals undergoing sham laparotomy.

Platelets have been shown to be one of the dominant contributors to hypercoagulability after injury (35). We therefore assessed the role of platelets in contributing to the systemic hypercoagulable state after local organ injury. Whole blood platelet aggregometry revealed that liver I/R led to a greater degree of systemic aggregation (Figure 2B; Supplementary Figure 1C). Though platelet counts remained consistent after liver I/R (Figure 2C; Supplementary Figure 2A), the platelet activation marker P-selectin (CD62P), was significantly increased in mice after liver I/R in comparison with sham mice (Figure 2D; Supplementary Figure 3A). P-selectin levels following I/R were time-dependent, with a peak level detected at 6 h after reperfusion.

Platelet-neutrophil aggregation is essential in immunothrombosis (36). With our initial studies demonstrating the presence of a systemic hypercoagulable state following liver I/R, we sought further to investigate the role of neutrophils as an effector of the innate immune response leading to platelet activation. To assess the interaction between the activated platelets and neutrophils, circulating platelet-neutrophil aggregates in whole blood were evaluated by flow cytometry. Liver I/R led to a significant and steady increase in circulating platelet-neutrophil aggregates, highlighting the importance of neutrophils and platelets in initiating a cascade that precipitates systemic hypercoagulability in the setting of a local liver insult (Figure 2E; Supplementary Figure 3B).

### Hepatic Surgical Stress Causes Local and Distant Organ Injury and Microvascular Immuno-Thrombi

**Figure 3:**
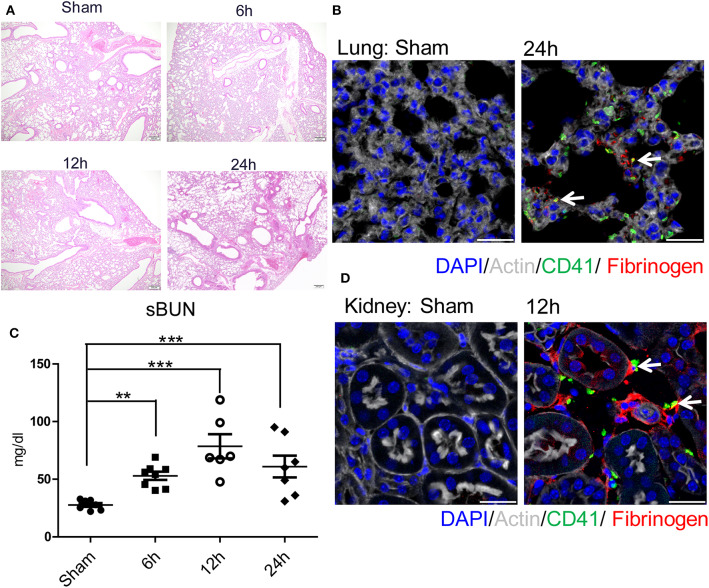
Hepatic surgical stress causes microvascular thrombi with organ injury. Compared with sham mice I/R leads to a **(A)** progressively increasing lung injury from 6/12/24 h reperfusion through histologic evaluation(4x); **(B)** more microvascular thrombi and fibrinogen rich areas on immunofluorescence imaging of the lung with staining for CD41 (green), nuclei (blue), fibrinogen(red), and Actin (white), scale bar, 25 μm; **(C)** increased serum BUN levels; **(D)** platelet accumulation within the glomeruli of kidney with staining for CD41 (green), nuclei (blue), fibrinogen(red), and Actin (white), scale bar, 25 μm (*n* = 6–8 each group). (Abbreviation: 6 h: ischemia 1.5 h followed by 6 h reperfusion; 12 h: ischemia 1.5 h followed by 12 h reperfusion; 24 h: ischemia 1.5 h followed by 24 h reperfusion). **p < 0.01 and ***p < 0.001.

We next sought to determine the mechanisms by which remote organs are impaired as a result of systemic hypercoagulability and platelet-neutrophil aggregates after liver I/R. A pattern of injury was seen in the lungs and the kidneys following liver I/R (Figures 3A,C; Supplementary Figures 3C,D). In addition, platelet accumulation, microvascular thrombi, and fibrinogen rich areas were identified in immunofluorescent imaging of both the lung and kidney (Figures 3B,D). These data indicated that liver I/R induces a pro-coagulant state and associated platelet-rich microvascular thrombi in distant organs.

Consistent with our previous finding, liver I/R led to severe damage in the liver with significantly increased serum ALT levels that peaked at 6 h after reperfusion and concurrent severe sinusoidal dilation and pericentral hepatocellular necrosis in the ischemic liver lobe (18). We also found that liver I/R led to a greater accumulation of platelets as well as the presence of microvascular fibrin thrombi within the sinusoids by labeling platelets and fibrinogen, as shown by immunofluorescent imaging (37) (Supplementary Figures 3E–G).

### Neutrophil Extracellular Traps (NETs) Are Involved in Local Remote Organ Injury After Hepatic Surgical Stress

**Figure 4:**
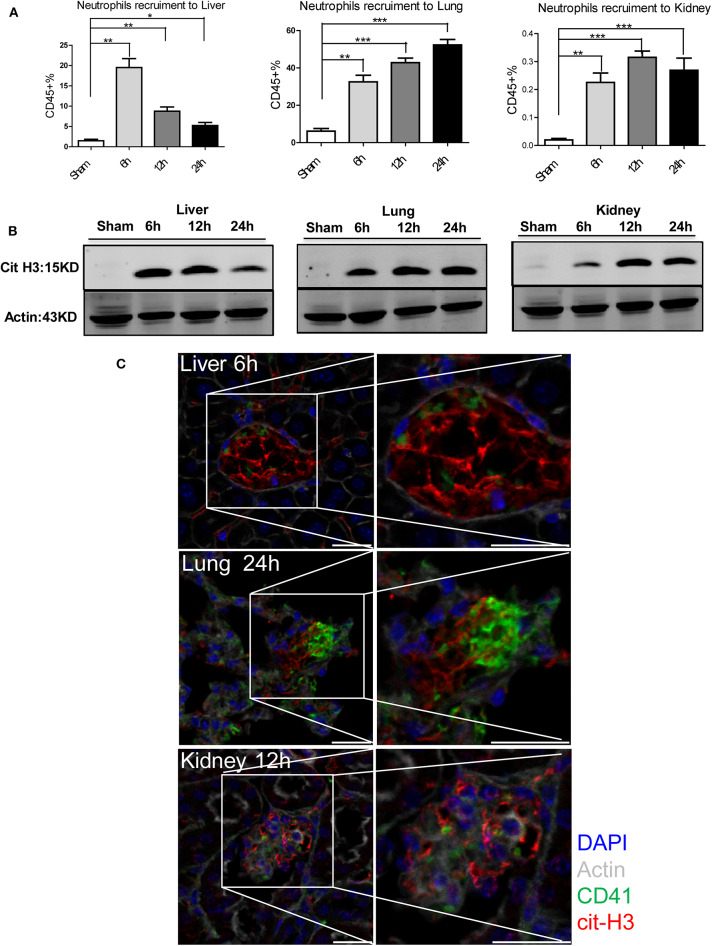
NETs are involved in systemic organ injury after hepatic stress. Compared with sham mice I/R leads to a **(A)** neutrophil migration into not only the liver but also into the lung and kidney; **(B)** NETs form after 1.5 h ischemia followed by 6, 12, 24 h of reperfusion in liver, lung and kidney as assessed by citrullinated-histone-3 protein levels were determined by western blot. **(C)** Immunofluorescence imaging(magnification ×20) reveals an increase co-localization of platelet and NET staining in liver, lung and kidney after I/R, with staining for CD41 (green), nuclei (blue), citrullinated histone H3 (cit-H3) (red), and Actin (white) (*n* = 4–6 in each group). Scale bar, 25 μm (Abbreviation: 6 h: ischemia 1.5 h followed by 6 h reperfusion; 12 h: ischemia 1.5 h followed by 12 h reperfusion; 24 h: ischemia 1.5 h followed by 24 h reperfusion). *p < 0.05, **p < 0.01 and ***p < 0.001.

We have previously demonstrated that NETs formed in the liver exacerbate hepatic injury after I/R (18). However, the effect of NETs on distant organ injury after liver I/R and their relationship to the pro-coagulant state are unknown. We therefore quantified neutrophil recruitment to liver, lung, and kidney by flow cytometry and found that liver I/R promoted neutrophil infiltration of not only the liver but also the lung and kidney (Figure 4A; Supplementary Figures 2B, 4A). Neutrophil infiltration peaked at 6, 24, and 12 h for liver, lung, and kidney, respectively. This sequence was concordant with the timing of the most severe biochemical and histological injury to each organ (Figure 3). We next assessed citrullinated-histone 3 (citH3), a marker of NETs (38), in liver, lung, and kidney. We found that citH3 levels are increased in all three organs after liver I/R, indicating that NETs are present even in distant organs after liver I/R (Figure 4B). In addition, immunofluorescence imaging revealed consistently increased NET staining co-localized with platelets in the liver, lung, and kidney (Figure 4C; Supplementary Figure 4B).

### Inhibiting NETs Mitigates Systemic Hypercoagulability and Microvascular-Thrombi in Distant Organs After Liver I/R

**Figure 5:**
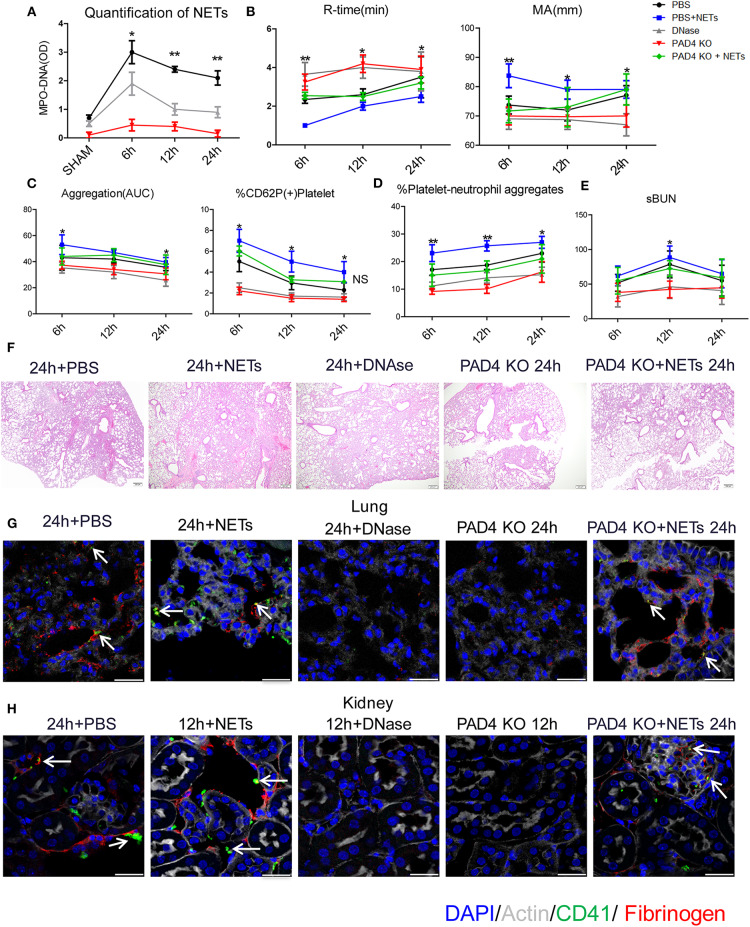
Inhibiting NETs protects against systemic microvascular-thrombi mediated organ injury after liver I/R. DNAse treatment or using PAD4^−/−^ mice in the I/R decreased **(A)** NETs formation by serum levels of MPO-DNA; extend **(B)** the time of clot formation, decrease the strength of clot formation; reduce **(C)** the rate of whole blood aggregation, platelet activation and **(D)** circulating platelet neutrophil aggregates; **(E)** serum BUN levels; **(F)** lung injury by histology; **(G)** microvascular thrombi and fibrinogen rich areas on immunofluorescence imaging of the lung with staining for CD41 (green), nuclei (blue), fibrinogen(red), and Actin (white), scale bar, 25 μm; **(H)** platelet accumulation within the glomeruli of kidney with staining for CD41 (green), nuclei (blue), fibrinogen(red), and Actin (white), scale bar, 25 μm. There is no significant difference between PAD4^−/−^ and DNase group, and treated PAD4^−/−^ mice with exogenous isolated NETs, which was comparable to the effects seen in PBS injection mice with systemic hypercoagulable status, local and distant organ injury of liver, lung and kidney result (*n* = 6–8 each group). (Abbreviation: 6 h: ischemia 1.5 h followed by 6 h reperfusion; 12 h: ischemia 1.5 h followed by 12 h reperfusion; 24 h: ischemia 1.5 h followed by 24 h reperfusion). *p < 0.05 and **p < 0.01.

To assess the role of NETs in systemic coagulation effects after liver I/R, we applied two strategies to block NETs: injection of DNase to disrupt NET fibers or utilizing PAD4^−/−^ mice (PAD4 key enzyme required for NET formation) (18, 22). In comparison to PBS injection group, DNase treatment significantly decreased neutrophil infiltration and formation of NETs not only in liver, lungs and kidneys but also in the circulation after liver I/R (Figure 5A; Supplementary Figures 5A,B). Similarly, PAD4^−/−^ mice subjected to liver I/R also exhibited decreased NET formation compared to control mice compared with PBS injection mice (Figure 5A). Blocking NETs by DNase treatment or using PAD4^−/−^ mice ameliorated the hypercoagulable status in mice as measured by R-time and MA. In contrast, mice treated with exogenous isolated NETs had a significantly shorter R-time and stronger clot formation (MA) compared with PBS injection control mice (Figure 5B). Activation of platelets and circulating platelet-neutrophil aggregates were reduced in DNase-treated and PAD4^−/−^ mice after liver I/R but increased when treated with exogenous isolated NETs by contrast with PBS injection mice (Figures 5C,D).

Similar to the effects seen with systemic hypercoagulation, local and distant organ injury of liver, lung and kidney were reduced with NET inhibition (Figures 5E,F; Supplementary Figures 6A–C) but aggravated when treated with exogenous isolated NETs in comparison to PBS injection group. Platelet rich microvascular immune-thrombi staining with CD41 and fibrinogen, seen in the lung and kidney after liver I/R, were drastically reduced in animals after blockade of NET formation compared with PBS injection control mice (Figures 5G,H; Supplementary Figure 6D). There is no significant difference between PAD4^−/−^ and DNase group. We therefore, treated PAD4^−/−^ mice with exogenous isolated NETs, which was comparable to the effects seen in PBS injection mice with systemic hypercoagulable status, local and distant organ injury of liver, lung and kidney result (Figures 5B–H). These findings suggest that NETs are required for induction of the systemic pro-coagulant state and subsequent microvascular immuno-thrombi seen in distant organ damage after liver I/R and the NETs formation is the key deficiency in PAD4^−/−^ mice responsible for the subsequent events in remote organ injury through microvascular immuno-thrombi.

### NETs Activate Platelets Through Platelet TLR4

**Figure 6:**
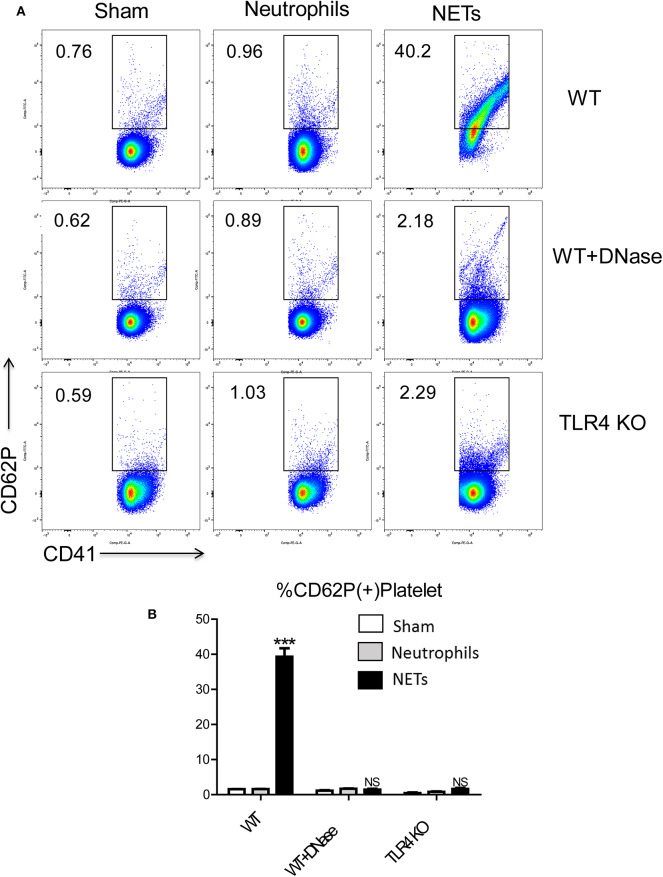
NET activation of platelets is dependent on platelet TLR4 signaling. Platelet from WT mice incubated with the medium from isolated NETs expressed **(A,B)** more CD62P than incubated with medium from unstimulated neutrophils; Platelet from TLR4 KO mice or with DNase treatment reduced platelet activation (*n* = 6–12 each group). (Abbreviation: PMA: Phorbol 12-myristate 13-acetate, PMN: polymorphonuclear). ***p < 0.001.

To determine the mechanisms by which NET might interact with platelets to facilitate a hypercoagulable state, we isolated bone marrow-derived neutrophils from WT mice and stimulated them *in vitro* to form NETs with phorbol 12-myristate 13-acetate (PMA). The media was collected and incubated with isolated WT platelets. Flow cytometry showed that platelets had significantly higher expression of surface CD62P, indicative of greater platelet activation when incubated with the media from stimulated neutrophils in comparison to incubation with media from unstimulated neutrophils (Figures 6A,B). Since PMA is known to affect platelet activation (28), PMA-free isolated NETs were used, and flow cytometry revealed pure NETs significantly increased platelet activation as well, this effect will be blocked by using DNase (Figures 6A,B). This data suggests that the cellular remnants and insoluble chromatin strands from NETs directly activate platelets.

Platelet TLR4 has been shown to be an essential receptor that mediates the coagulation response in sterile inflammation such as hemorrhagic shock and resuscitation (23). It has been reported that platelet TLR4 can activate blood neutrophils during sepsis to form NETs in order to ensnare bacteria (39). However, whether the converse of NETs activating platelets through platelet TLR4 has not been described. We therefore, obtained platelets from TLR4 KO or WT mice and incubated them with isolated NETs, in addition with DNase or control. Isolated NETs failed to activate TLR4 KO platelets, indicated by the reduced number of P-selectin-CD41 double-positive cells compared with WT platelets, suggesting that both TLR4 on platelets are necessary for platelet activation *in vitro* (Figures 6A,B).

### TLR4 Depletion on Platelets Confers Protection of Liver and Remote Organs From Liver I/R

**Figure 7:**
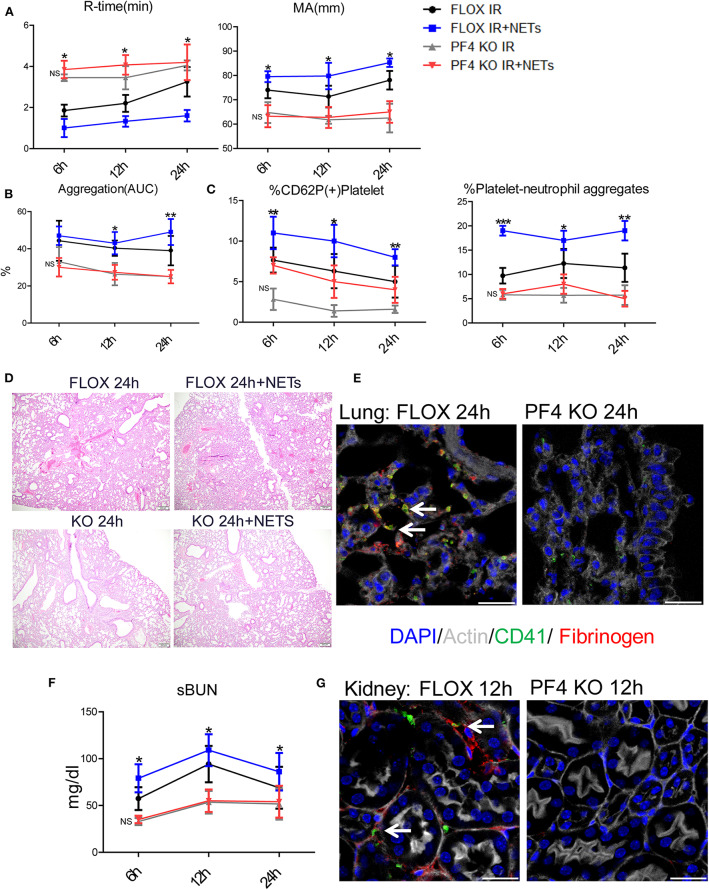
Blocking NET-platelet interaction by TLR4 protects against systemic immunothrombosis effects. Compared with FLOX mice, TLR4loxP/Pf4-Cre mice undergoing liver IR reduced **(A)** extend the time of clot formation, decrease the strength of clot formation; shorter time to clot formation (R-time) and increased clot strength (MA) in Flox mice treated with PMA-free isolated NETs whereas no significant difference in PF4 TLR4 KO mice when treated with PMA-free isolated NETs after liver I/R; Compared with FLOX mice, TLR4loxP/Pf4-Cre mice undergoing liver IR reduced **(B)** the rate of whole blood aggregation, platelet activation; **(C)** circulating platelet neutrophil aggregates; **(D)** lung injury by histology; **(E)** microvascular thrombi and fibrinogen rich areas on immunofluorescence imaging of the lung with staining for CD41 (green), nuclei (blue), fibrinogen(red), and Actin (white), scale bar, 25 μm; **(F,G)** serum BUN levels and platelet accumulation within the glomeruli of kidney with staining for CD41 (green), nuclei (blue), fibrinogen(red), and Actin (white), scale bar, 25 μm. Organs injury and hypercoagulable state increased in Flox mice treated with PMA-free isolated NETs whereas no significant difference in PF4 TLR4 KO mice when treated with PMA-free isolated (*n* = 6–8 each group). (Abbreviation: 6 h: ischemia 1.5 h followed by 6 h reperfusion; 12 h: ischemia 1.5 h followed by 12 h reperfusion; 24 h: ischemia 1.5 h followed by 24 h reperfusion; FLOX:TLR4loxP FLOX,PF4 KO:TLR4loxP/Pf4-Cre). *p < 0.05, **p < 0.01 and ***p < 0.001.

To determine if NETs can activate platelets after hepatic I/R *in vivo* through platelet-TLR4, we utilized PF4 TLR4 KO (TLR4^loxP/Pf4−Cre^) mice, in which *tlr4* is specifically knocked out in platelet. We observed that PF4 TLR4 KO mice exhibited an amelioration of the pro-coagulant state as measured by R-time and MA in TEG analysis compared to control Flox mice (Figure 7A). A pro-coagulant state was demonstrated in Flox control mice treated with exogenous isolated NETs but there was no significant difference in PF4 TLR4 KO mice when treated with or without exogenous isolated NETs after liver I/R (Figure 7A). Platelet rate of aggregation, maximum aggregation, and activation of platelets were all significantly reduced in the PF4 TLR4 KO mice compared to their Flox counterparts (Figure 7B). These findings were consistent with less circulating platelet-neutrophil aggregates as well in PF4 TLR4 KO mice (Figure 7C).

PF4 TLR4 KO mice also had decreased microvascular thrombi and damage in the lung, kidney, and liver in comparison to their Flox counterpart controls (Figures 7E,G; Supplementary Figure 7D). The damage in these organs was increased in Flox mice treated with exogenous isolated NETs whereas there was no significant difference in PF4 TLR4 KO mice when treated with or without isolated NETs after liver I/R (Figures 7D,F; Supplementary Figures 7A–C). Interestingly, we found that there are no differences of NETs expression in the organs after liver I/R in Flox mice compared with PF4 TLR4 KO mice, indicating that NETs coming first and activate TLR4 on platelet (Supplementary Figure 7E). Taken together, in comparison to Flox controls mice, PF4 TLR4 KO mice had decreased organs injury and hypercoagulable state, Flox mice treated with exogenous isolated NETs increased organs damage and hypercoagulable state. Whereas, there is no significant difference between PF4 TLR4 KO mice when treated with or without isolated NETs.

In order to address concerns of PF4-Cre inducing recombination in various hematopoietic cell types beyond megakaryocytes (40, 41), we repeated our experiments using thrombocytopenic mice. Native platelets from WT (TLR4^loxp/loxp^) and PF4 TLR4 KO mice were depleted using an antiplatelet antibody. Platelets were then harvested from either WT or TLR4 global KO mice and transfused into thrombocytopenic recipients. Native platelets were < 10% of circulating platelets in the transfused recipients (data not shown). Interestingly, transfusion of TLR4(+) platelets into thrombocytopenic platelet TLR4(–) (PF4 TLR4 KO) mice reversed the reduced pro-coagulant status and resulted in a similar pro-coagulant state as shown in WT control [transfusion of TLR4(+) platelets into thrombocytopenic WT] mice. Transfusion of TLR4(–) platelets from TLR4 KO mice failed to initiate the pro-coagulant state seen in thrombocytopenic WT animals after I/R (Supplementary Figures 8A–C). Similar effects were also seen in the degree of organ injury (Supplementary Figures 8C–F). These findings suggest that platelet TLR4 is required for NET-mediated systemic pro-coagulant state and organ injury after liver I/R.

## Discussion

There is now increasing evidence that patients undergoing liver injury or surgery are actually hypercoagulable in the perioperative state rather than hypocoagulable from traditional concerns with decreased production of coagulation factors in the liver (42–44). This has important clinical implications such as re-evaluation of how to address perioperative venous thromboembolism prophylaxis after liver resection, transplantation, or trauma. Liver I/R injury is known to cause distant organ injury (16). However, the mechanism of this systemic postoperative pro-coagulant state with resultant distant organ injury is not well-understood. In this study we focused on innate immune responders, particularly neutrophils, since they play a fundamental role in the systemic effects of liver I/R (45, 46). Neutrophils and their formation of NETs impact a plethora of disease processes through intricate interactions with the coagulation system, an interaction now termed immunothrombosis (7, 47). Our findings demonstrate that liver I/R is associated with immunothrombosis, as evidenced by circulating platelet activation, platelet-neutrophil interactions, and platelet-rich microvascular thrombi both within the liver and remote organs. Additionally, we demonstrate that NETs are crucial for the formation of these platelet-rich microvascular thrombi since the inhibition of NETs ameliorates the pro-coagulant state and injury of multiple organs after liver I/R.

The interaction between NETs and platelets is vital in the initiation of immunothrombosis effects. Platelet TLR4 has been shown in infectious models to be a link where platelet TLR4 can activate neutrophils to form NETs in septic blood (39). Interestingly, this was not seen in our study as the levels of NET formation in organs after liver I/R were not different between platelet specific TLR4 KO and control mice (Supplementary Figure 7E). The discordance of these findings could possibly be a result of using different experimental animal models for initiation of inflammation (i.e., infectious vs. sterile). For example, we previously demonstrated that damage associated molecular patterns (DAMPs), such as exogenous HMGB1 (High Mobility Group Box 1) (48) and histones (49) released from damaged hepatocytes are the main activators to induce neutrophils to form NETs in the liver during liver I/R (18). Moreover, it is known that HMGB1 release from platelets enhances neutrophil recruitment and promotes NET formation that regulates thrombosis (50). There is rapidly growing evidence that Reactive Oxygen Species (ROS) are able to interact with the formation of ETs (Extracellular Traps) in a multidimensional manner (51, 52). And ROS are involved in the regulation of all of the major processes that promote the formation of venous thrombi which include coagulation; platelet reactivity; and sterile inflammation (for example NETosis) during formation (53). Thus, in our model, released DAMPs or ROS from the liver may be the primary stimulators to produce NETs rather than platelet TLR4 as seen during infection. NETs have also been shown to increase thrombosis in human blood *in vitro* through a TLR2 and TLR4 dependent mechanism (47, 54). Furthermore, histone-activated platelets possess a procoagulant phenotype through TLR2 and TLR4 mediated process which has also been shown in human blood *in vitro* (55). However, our finding is the first to demonstrate that NETs can activate platelets through platelet TLR4 *in vivo*. Our recent finding demonstrated that HMGB1 is involved in NETs (34), which might activate platelets through TLR4. This process can explain the systemic affects following local liver injury through the facilitation of small vessel platelet-rich thrombi formation in remotely organs.

Targeting NETs through DNase may provide therapeutic benefit in the clinical setting by preventing the wide-spread systemic effects that result from local injuries, particularly related to liver resection, transplantation, and shock. DNase has been shown to be beneficial in the treatment of other diseases with inflammatory pathways including cystic fibrosis and acute lung injury, and has few systemic adverse side effects (56, 57). DNase, which degrades NETs has been shown to protect mice from venous thrombosis (58). Importantly, defects of both DNases failed to degrade intravascular NET clots and contribute to vascular occlusions in patients with severe bacterial infections (59). However, the role of DNase in liver I/R in the clinical perioperative setting has not yet been determined. Our study demonstrate that DNase is an appealing treatment for hepatic I/R and its systemic effects as there are few alternative measures to prevent the widespread effects of liver I/R. The specificity of DNase treatment for NETs is an area of active investigation as DNase most likely has protective effects beyond NET degradation since it can digest other extracellular DNA complexes released from dead cells. Furthermore, we genetically block NET formation using PAD4^−/−^ mice, which are incapable of forming NETs (22, 34). In our model, blocking NETs by DNase treatment or using PAD4^−/−^ mice ameliorated the systemic hypercoagulation, local and distant organ injury and treating PAD4^−/−^ mice with exogenous isolated NETs, which was comparable to the effects seen in PAD4 WT mice with systemic hypercoagulable status, local and distant organ injury of liver, lung and kidney, which demonstrates that the NET formation was the key deficiency in PAD4 KO mice responsible for the subsequent events in remote organ injury through microvascular immuno-thrombi. Our data support the hypothesis that NETs are required for induction of the systemic pro-coagulant state and subsequent microvascular immuno-thrombi seen in distant organ damage after liver I/R.

In conclusion, our study demonstrates that liver I/R leads to a NET-mediated hypercoagulable state and subsequent remote organ injury through microvascular immuno-thrombi. The effects of NETs are mediated through platelet TLR4-dependent pathways. These findings give promise to a potential therapeutic approach targeting NETs with DNase that can not only be protective against the local effects of liver I/R but also for treating and preventing a systemic hypercoagulable state and further remote organ injury.

## Data Availability Statement

The datasets generated for this study are available on request to the corresponding author.

## Ethics Statement

The studies involving human participants were reviewed and approved by University of Pittsburgh Medical Center (Pittsburgh, PA). All human materials were obtained under an approved Institutional Review Board protocol (MOD08010372-25/PRO08010372). The patients/participants provided their written informed consent to participate in this study. The animal study was reviewed and approved by Animal Care and Use Committee of the University of Pittsburgh.

## Author Contributions

HZ, JG, AT, and HH: conception and design. HZ, JG, PL, AT, and HH: development of methodology. JG, PV, DW, JR, PL, and HH: acquisition of data (provided animals, acquired and managed patients, provided facilities, etc.). HZ, JG, PV, DW, JR, and HH: analysis and interpretation of data (e.g., statistical analysis, biostatistics, computational analysis). HZ, JG, MN, RS, AT, and HH: writing, review, and/or revision of the manuscript. JR and HY: administrative, technical, or material support (i.e., reporting or organizing data, constructing databases). JZ, AT, and HH: study supervision.

## Conflict of Interest

The authors declare that the research was conducted in the absence of any commercial or financial relationships that could be construed as a potential conflict of interest.
