# Supplementary material for: Hepatic Surgical Stress Promotes Systemic Immunothrombosis That Results in Distant Organ Injury
Source: Front Immunol. 2020 May 22;11:987. doi: 10.3389/fimmu.2020.00987 (PMC7256968; doi:10.3389/fimmu.2020.00987)
Supplement: Supplementary file 1 [file Presentation_1.PPTX]

## Slide 1
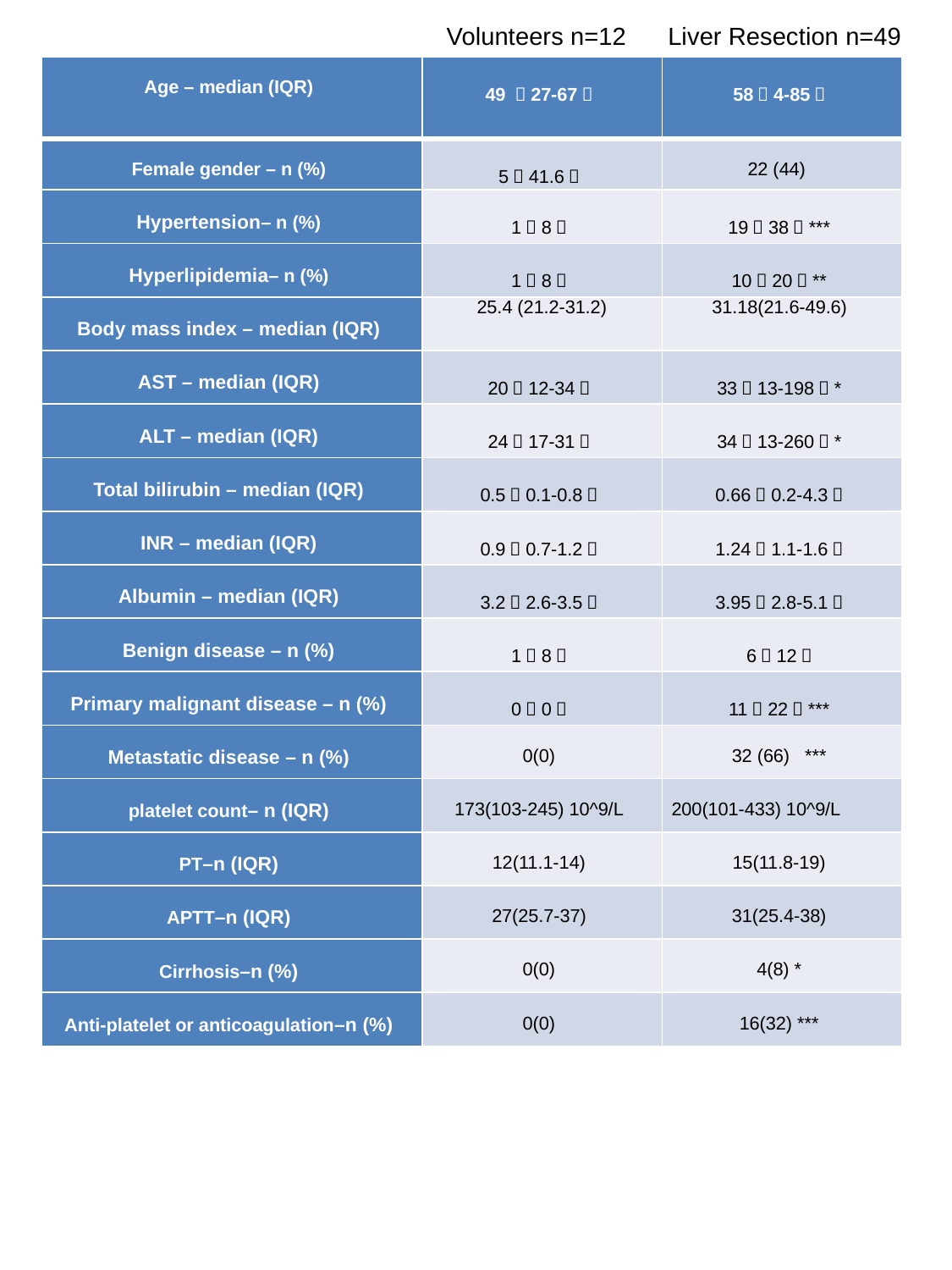

Volunteers n=12 Liver Resection n=49
| Age – median (IQR) | 49 （27-67） | 58（4-85） |
| --- | --- | --- |
| Female gender – n (%) | 5（41.6） | 22 (44) |
| Hypertension– n (%) | 1（8） | 19（38）\*\*\* |
| Hyperlipidemia– n (%) | 1（8） | 10（20）\*\* |
| Body mass index – median (IQR) | 25.4 (21.2-31.2) | 31.18(21.6-49.6) |
| AST – median (IQR) | 20（12-34） | 33（13-198）\* |
| ALT – median (IQR) | 24（17-31） | 34（13-260）\* |
| Total bilirubin – median (IQR) | 0.5（0.1-0.8） | 0.66（0.2-4.3） |
| INR – median (IQR) | 0.9（0.7-1.2） | 1.24（1.1-1.6） |
| Albumin – median (IQR) | 3.2（2.6-3.5） | 3.95（2.8-5.1） |
| Benign disease – n (%) | 1（8） | 6（12） |
| Primary malignant disease – n (%) | 0（0） | 11（22）\*\*\* |
| Metastatic disease – n (%) | 0(0) | 32 (66) \*\*\* |
| platelet count– n (IQR) | 173(103-245) 10^9/L | 200(101-433) 10^9/L |
| PT–n (IQR) | 12(11.1-14) | 15(11.8-19) |
| APTT–n (IQR) | 27(25.7-37) | 31(25.4-38) |
| Cirrhosis–n (%) | 0(0) | 4(8) \* |
| Anti-platelet or anticoagulation–n (%) | 0(0) | 16(32) \*\*\* |

## Slide 2
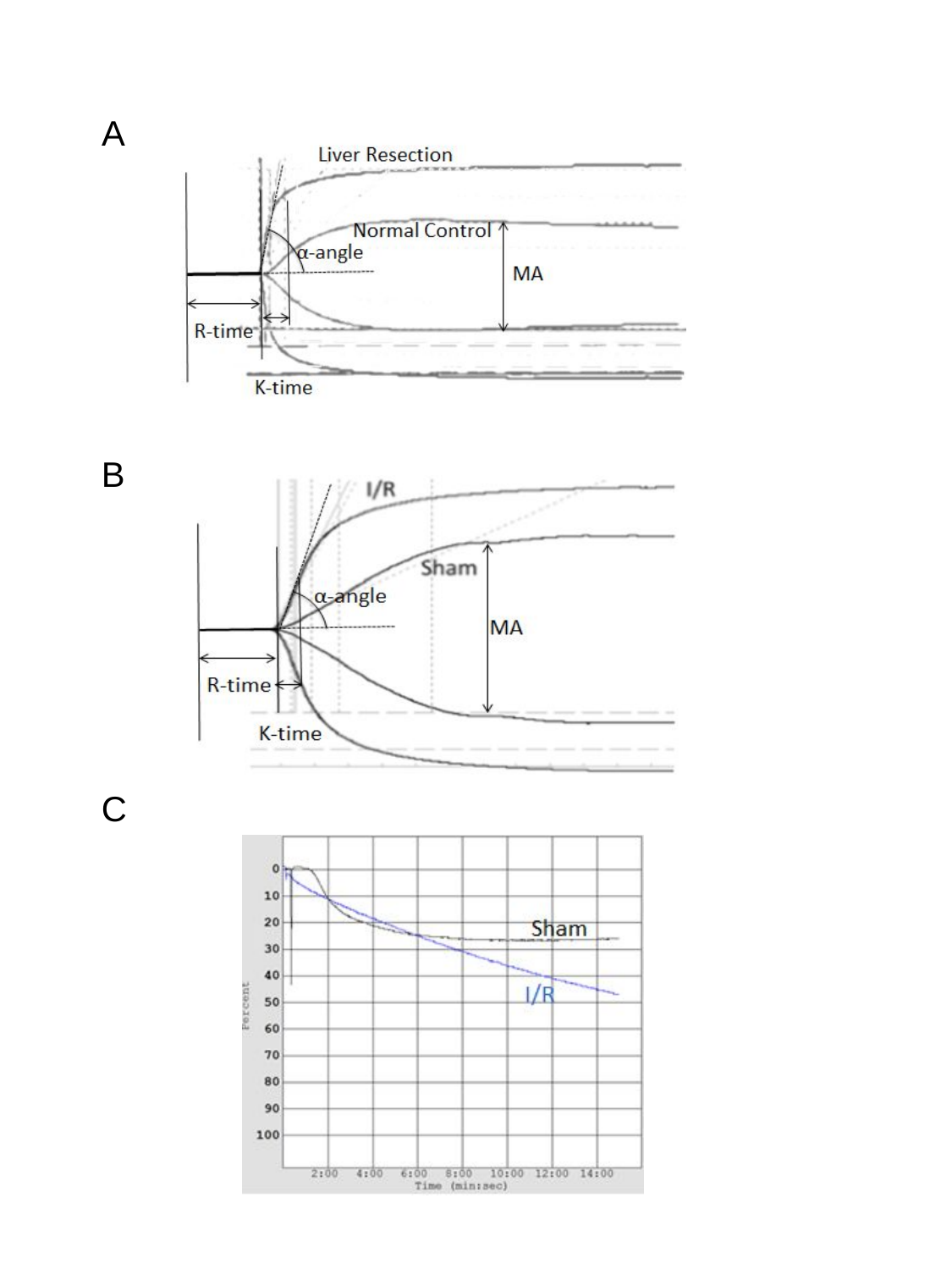

A
B
C

## Slide 3
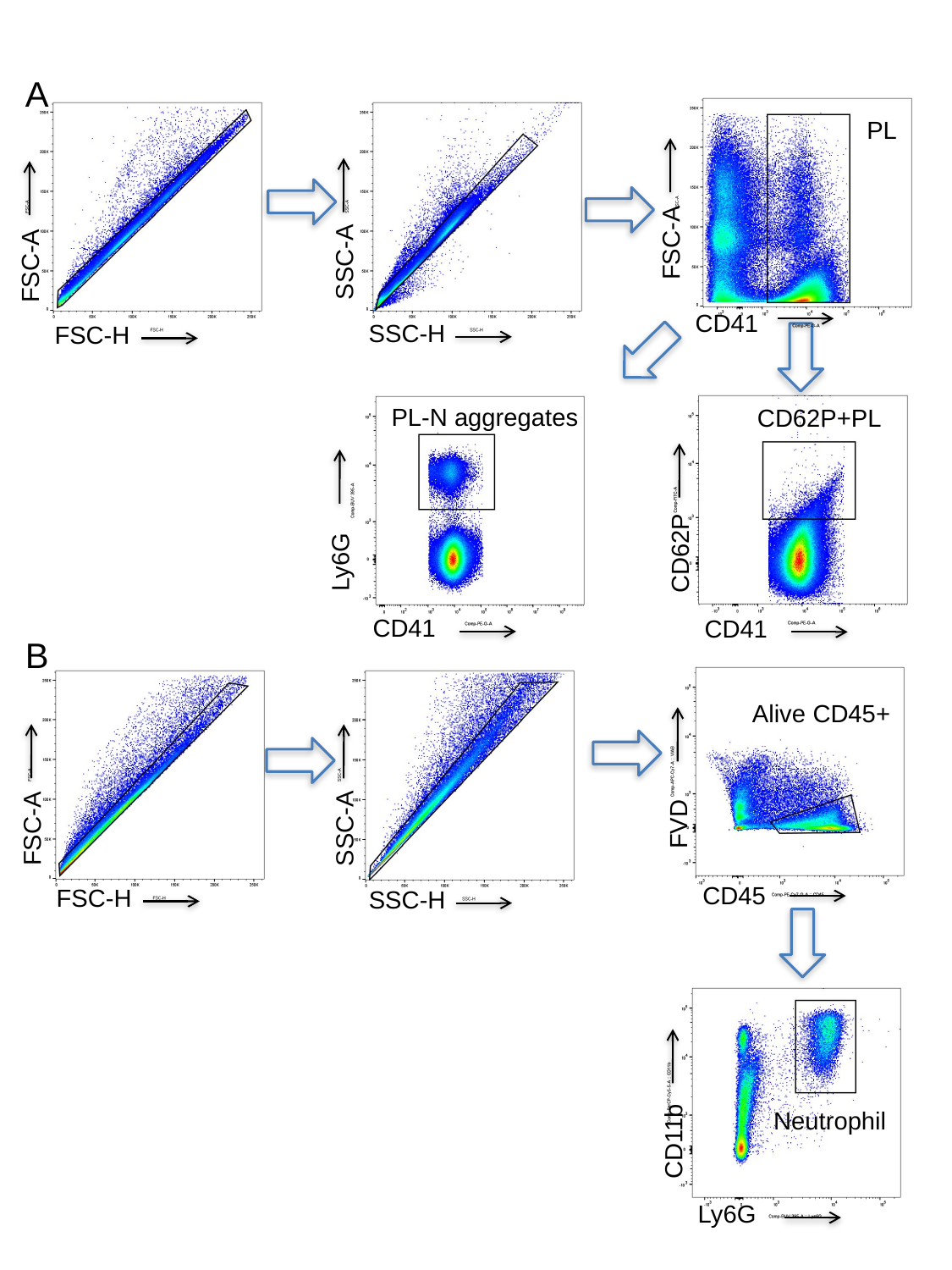

A
FSC-A
CD41
PL
FSC-A
FSC-H
SSC-A
SSC-H
CD62P
CD41
CD62P+PL
Ly6G
CD41
PL-N aggregates
B
FSC-A
FSC-H
FVD
CD45
SSC-A
SSC-H
Alive CD45+
CD11b
Ly6G
Neutrophil

## Slide 4
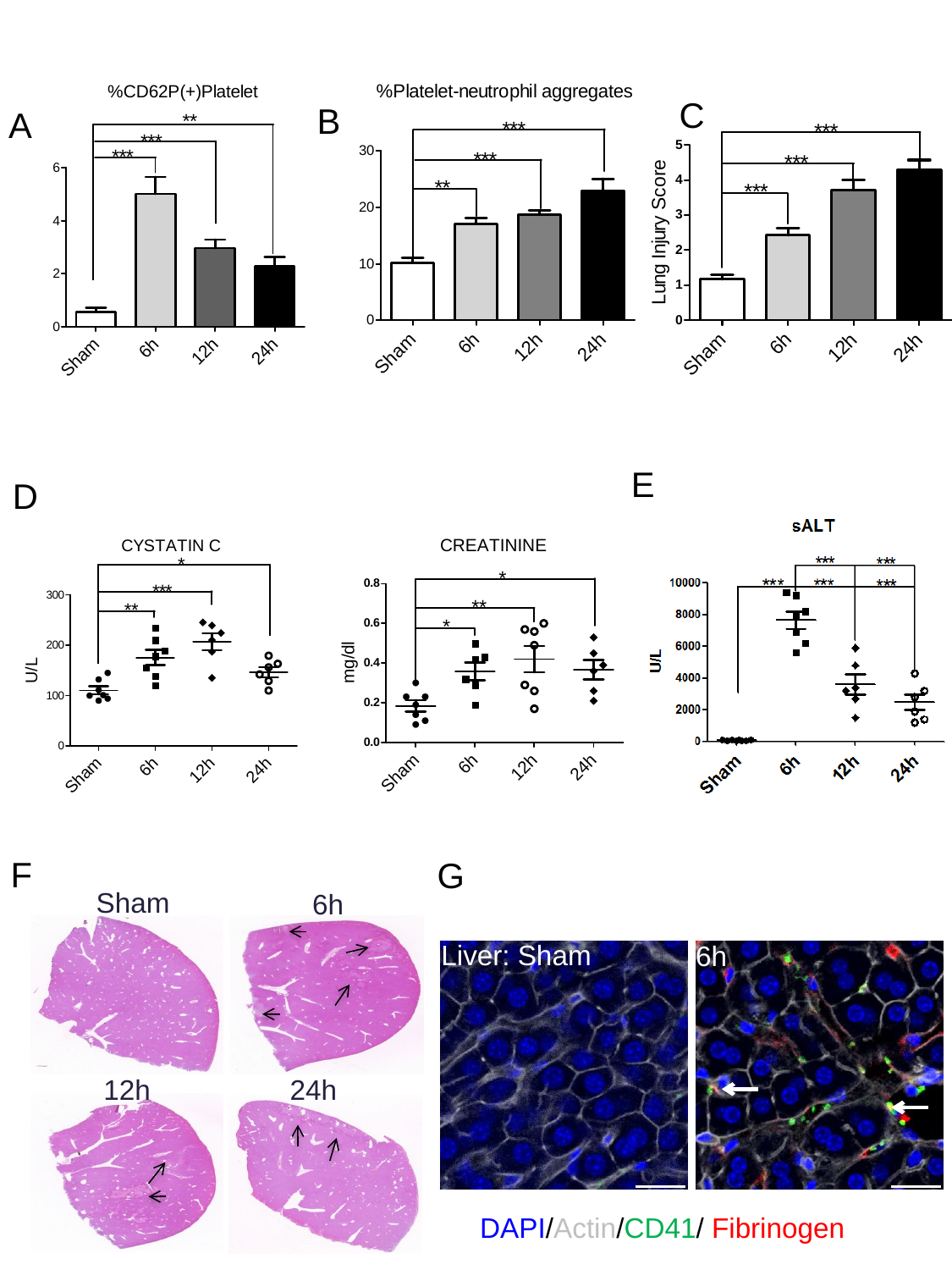

C
B
A
E
D
F
G
Sham
6h
12h
24h
Liver: Sham
6h
DAPI/Actin/CD41/ Fibrinogen

## Slide 5
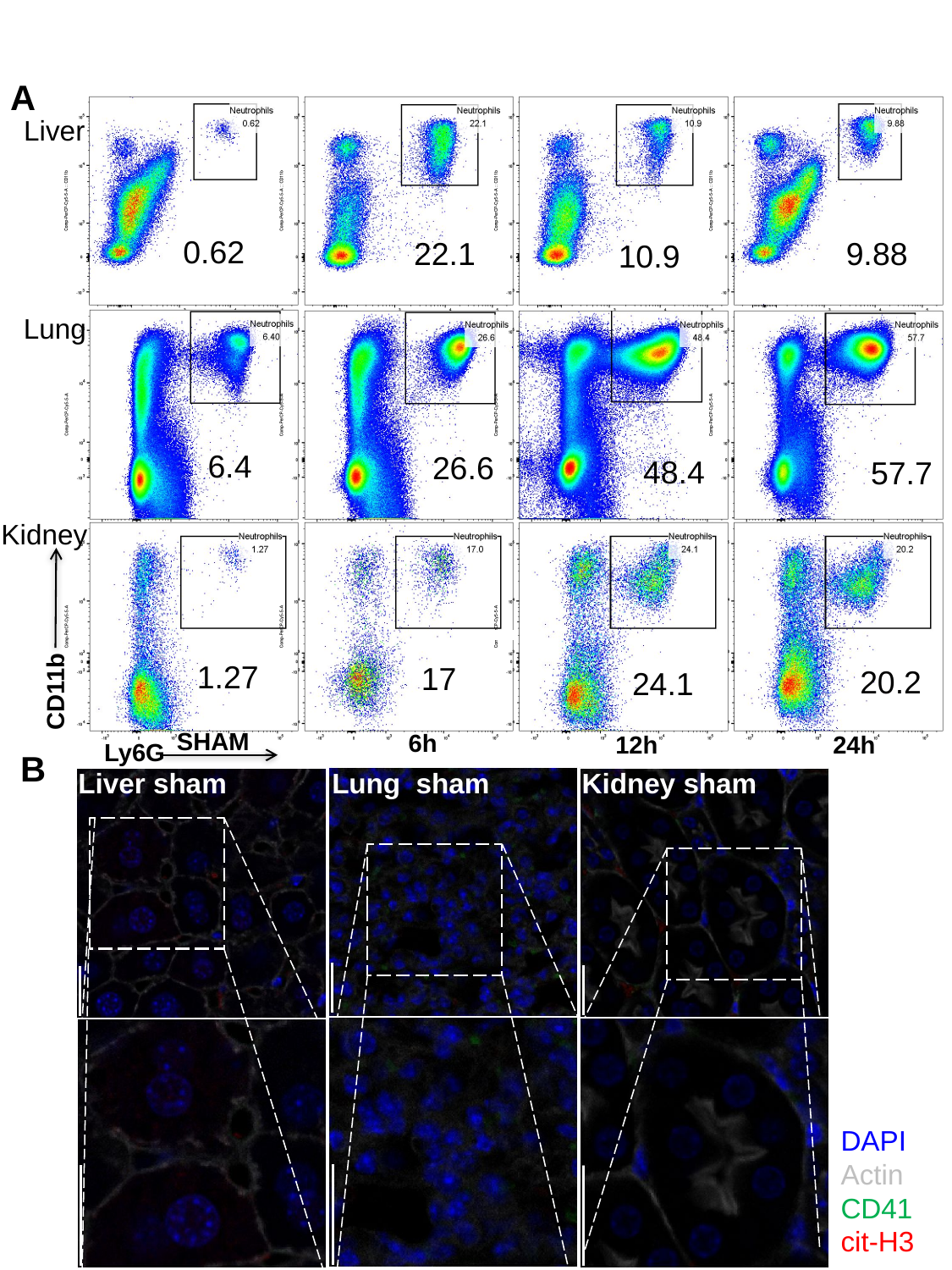

A
0.62
9.88
22.1
10.9
Liver
6.4
26.6
48.4
57.7
Lung
Kidney
1.27
17
20.2
24.1
CD11b
SHAM
6h
12h
24h
Ly6G
B
Liver sham
Kidney sham
Lung sham
DAPI
Actin
CD41
cit-H3

## Slide 6
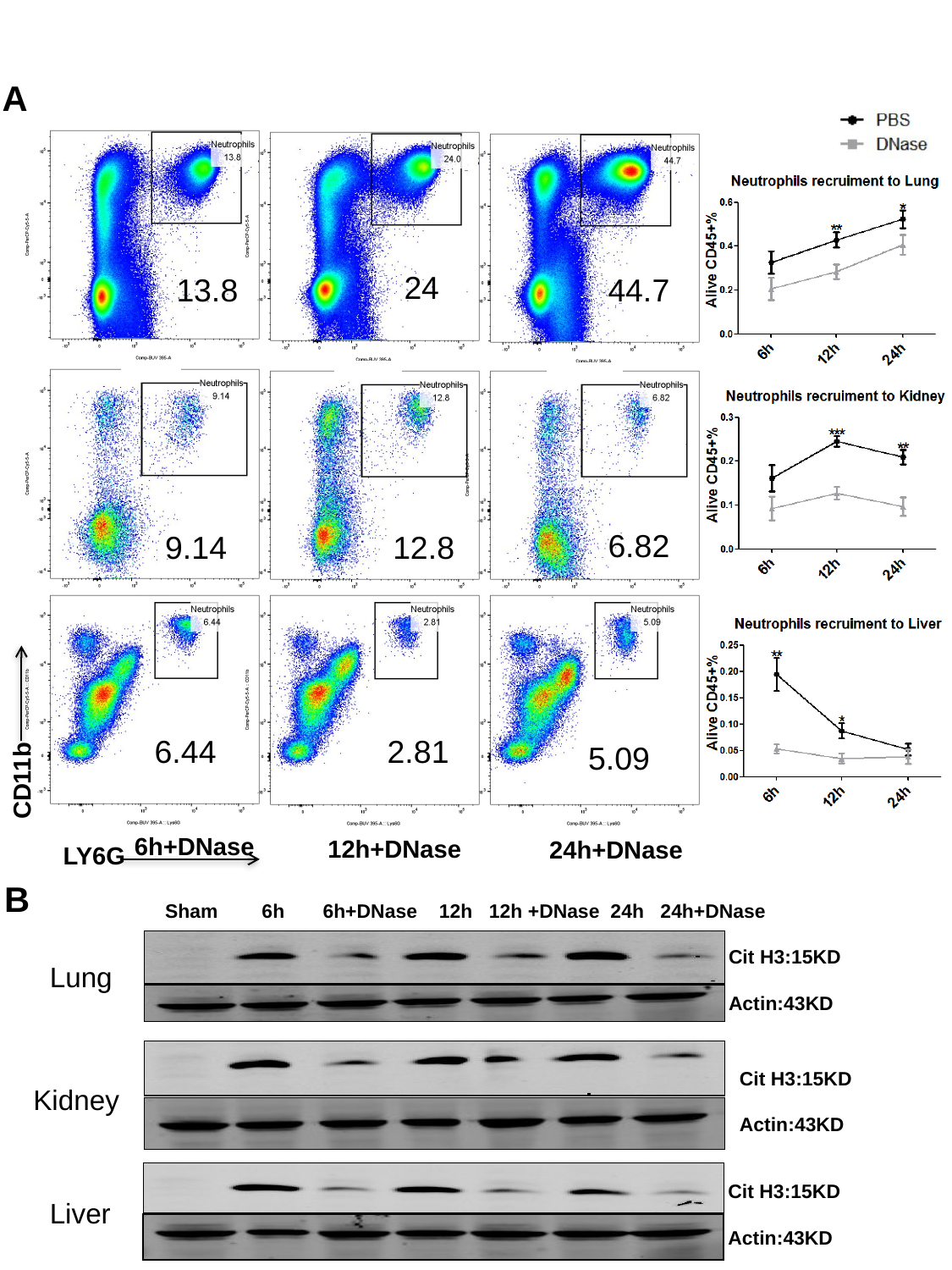

A
24
13.8
44.7
9.14
12.8
CD11b
6h+DNase
12h+DNase
24h+DNase
LY6G
6.82
6.44
2.81
5.09
 Sham 6h 6h+DNase 12h 12h +DNase 24h 24h+DNase
Cit H3:15KD
Actin:43KD
Lung
 Cit H3:15KD
 Actin:43KD
Kidney
Cit H3:15KD
Actin:43KD
Liver
B

## Slide 7
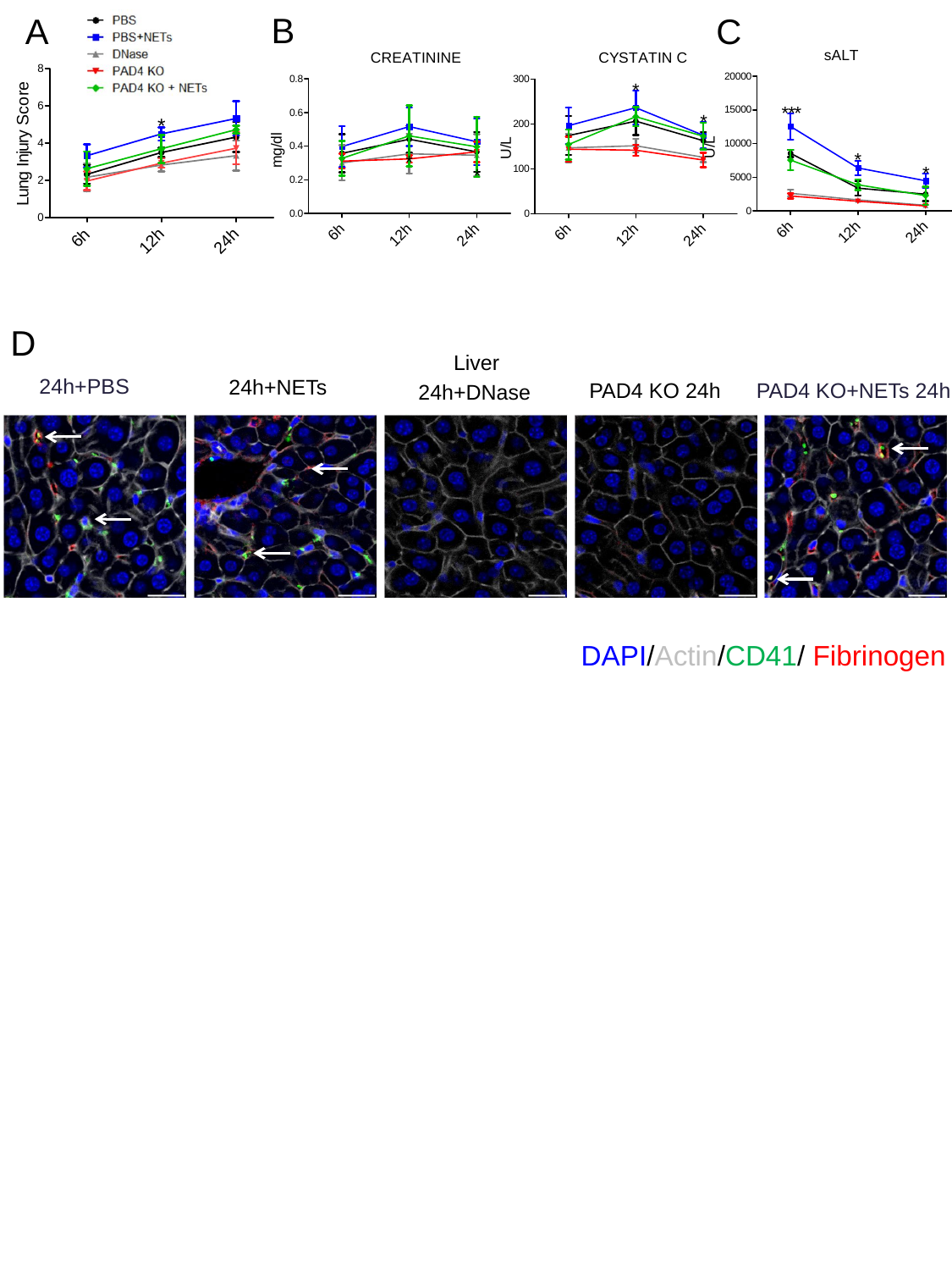

B
A
C
D
Liver
24h+PBS
24h+NETs
PAD4 KO 24h
PAD4 KO+NETs 24h
24h+DNase
DAPI/Actin/CD41/ Fibrinogen

## Slide 8
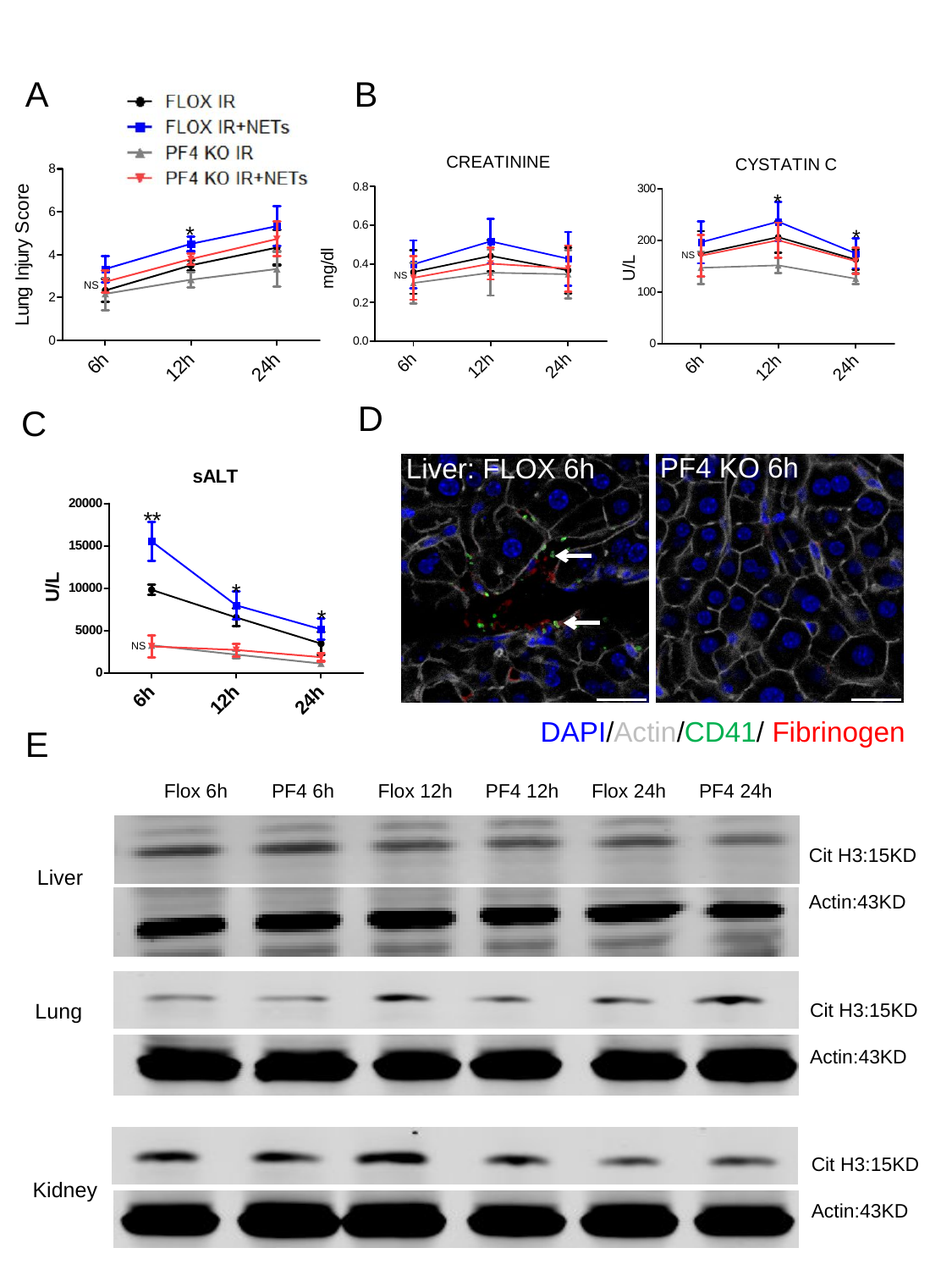

A
B
D
C
PF4 KO 6h
Liver: FLOX 6h
DAPI/Actin/CD41/ Fibrinogen
E
 Flox 6h PF4 6h Flox 12h PF4 12h Flox 24h PF4 24h
Cit H3:15KD
Actin:43KD
Lung
Cit H3:15KD
Actin:43KD
Kidney
Cit H3:15KD
Actin:43KD
Liver

## Slide 9
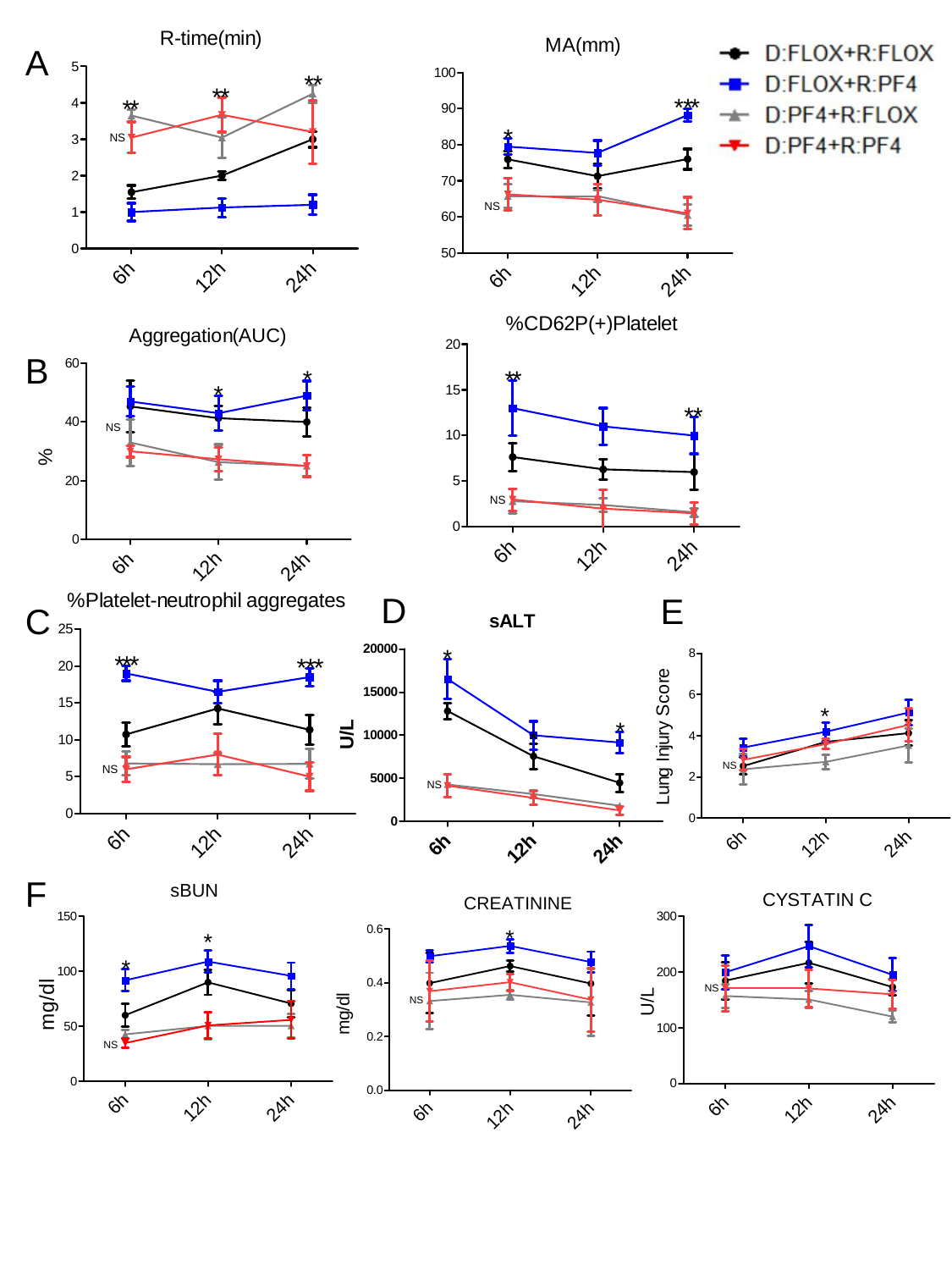

A
PF4 KO 6h
B
D
E
C
F
